# Maximizing the Utility of Alzheimer’s Disease Trial Data: Sharing of Baseline A4 and LEARN Data

**DOI:** 10.14283/jpad.2024.120

**Published:** 2024-07-24

**Authors:** Gustavo A. Jimenez-Maggiora, A. P. Schulz, M. C. Donohue, H. Qiu, S. N. Jaiswal, O. Adegoke, R. Gallardo, O. Baryshnikava, R. A. Rissman, S. Abdel-Latif, R. A. Sperling, P. S. Aisen, A4 and LEARN Study Teams

**Affiliations:** 1 Alzheimer’s Therapeutic Research Institute, University of Southern California, 9860 Mesa Rim Road, San Diego, CA 92121 USA; 2 Center for Alzheimer Research and Treatment, Brigham and Women’s Hospital, Massachusetts General Hospital, Harvard Medical School, Boston, MA USA; 3 https://www.actcinfo.org/a4-study-team-lists/

**Keywords:** Alzheimer’s disease, clinical trials, data sharing, open science

## Abstract

**Background:**

The Anti-Amyloid Treatment in Asymptomatic Alzheimer’s Disease (A4) and Longitudinal Evaluation of Amyloid Risk and Neurodegeneration (LEARN) studies were conducted between 2014 and 2023, with enrollment completed in 2017 and final study results reported in 2023. The study screening process involved the collection of initial clinical, cognitive, neuroimaging, and genetic measures to determine eligibility. Once randomized, enrolled participants were assessed every four weeks over a 4.5-year follow-up period during which longitudinal clinical, cognitive, and neuroimaging measures were collected. A large number of longitudinal fluid biospecimens were also collected and banked. Consistent with the NIH data sharing policy and the principles of Open Science, the A4/LEARN investigators aimed to share data as broadly and early as possible while still protecting participant privacy and confidentiality and the scientific integrity of the studies.

**Objectives:**

We describe the approach, methods, and platforms used to share the A4 and LEARN pre-randomization study data for secondary research use. Preliminary results measuring the impact of these efforts are also summarized. We conclude with a discussion of lessons learned and next steps.

**Design:**

The materials shared included de-identified quantitative and image data, analysis software, instruments, and documentation.

**Setting:**

The A4 and LEARN Studies were conducted at 67 clinical trial sites in the United States, Canada, Japan, and Australia.

**Participants:**

The A4 study screened (n=6763), enrolled, and randomized (n=1169) participants between the ages of 65 and 85 with a blinded follow-up period of 240 weeks followed by an open-label period of variable length. The LEARN study screened and enrolled individuals (n=538) who were ineligible for the A4 study based on nonelevated measures of amyloid accumulation using positron emission tomography imaging (amyloid PET).

**Measurements:**

We provide descriptive measures of the data shared and summarize the frequency, characteristics, and status of all data access requests submitted to date. We evaluate the scientific impact of the data-sharing effort by conducting a literature search to identify related publications.

**Results:**

The A4 and LEARN pre-randomization study data were released in December 2018. As of May 8, 2024, 1506 requests have been submitted by investigators and citizen scientists from more than 50 countries. We identified 49 peer-reviewed publications that acknowledge the A4/LEARN study.

**Conclusions:**

Our initial results provide evidence supporting the feasibility and scientific utility of broad and timely sharing of Alzheimer’s disease trial data.

## Introduction

The Anti-Amyloid Treatment in Asymptomatic Alzheimer’s Disease (A4) study (ClinicalTrials. gov ID: NCT02008357) was a phase 3 randomized, double-blind, placebo-controlled, parallelarm clinical trial evaluating the slowing of cognitive symptoms in pre-symptomatic Alzheimer’s participants treated with a monoclonal antibody, Solanezumab, versus placebo (1). This study screened (n=6763), enrolled, and randomized (n=1169) participants between the ages of 65 and 85 with a blinded follow-up period of 240 weeks followed by an open-label period of variable length. The study was conducted between 2014 and 2023, with enrollment completed in 2017 and final study results reported in 2023 (1, 2). The study screening process involved the collection of initial clinical, cognitive, neuroimaging, and genetic measures to determine eligibility. Once randomized, enrolled participants were assessed every four weeks over a 4.5-year follow-up period during which longitudinal clinical, cognitive, and neuroimaging measures were collected. A large number of longitudinal fluid biospecimens were also collected and banked.

A sister study, the LEARN (Longitudinal Evaluation of Amyloid Risk and Neurodegeneration) natural history cohort (ClinicalTrials.gov ID: NCT02488720), screened and enrolled individuals (n=538) who were ineligible for the A4 study based on nonelevated measures of amyloid accumulation using positron emission tomography imaging (amyloid PET). These participants were assessed longitudinally using a visit schedule and measures similar to the A4 study.

As an NIH-funded program, the A4 study was required to comply with NIH data-sharing policies (3, 4). These policies define the expectations of funded investigators regarding sharing their final research data, including providing a data-sharing plan, timelines for releasing the final data, and annual reporting requirements. However, from the earliest stages of study planning, the A4/LEARN investigators expressed a commitment to go beyond these minimum requirements in accordance with the Collaboration for Alzheimer’s Prevention guidelines (5), aiming to release data as broadly and early as possible while still protecting participant privacy and confidentiality and the scientific integrity of the studies. The A4/LEARN investigators proposed to release study data in two tranches based on the completion of study milestones: 1) release all screening and pre-randomization baseline data one year after the completion of study enrollment, and 2) release final data one year after the completion of the study.

The scope of the materials to be shared included de-identified quantitative and image data, analysis software, instruments, and documentation. The ultimate goal of the A4/LEARN investigators was to share data broadly to maximize the scientific utility of the generous contributions made by participants and study partners, study sponsors, study sites, and the public.

Central to this plan was the need to balance competing requirements of protecting participant privacy and confidentiality, protecting the scientific integrity of the trial, and meeting legal and regulatory obligations while sharing a broad set of data. Establishing and maintaining equipoise between the related benefits and risks of this approach required a collaborative planning and decision-making process that included input from a broad set of stakeholders.

The initial tranche of screening and pre-randomization data was released at the end of 2018, followed by an update that included baseline cross-sectional neuroimaging data released in early 2020. The purpose of this article is to describe the approach, methods, and platforms used to share the A4 and LEARN pre-randomization study data for secondary research use. Preliminary results measuring the impact of these efforts are also summarized. We conclude with a discussion of lessons learned and next steps.

## Methods

### Data Sharing Approach

Our approach to data sharing began with the development of a data-sharing plan. During initial planning, input was gathered from multiple study stakeholders, including the principal investigators, study sponsors, and experts in biostatistics, biomedical informatics, biomarkers and biobanking, neuroimaging, genetics, legal and regulatory compliance. External advisors were consulted on specific topics, such as appropriate methods for sharing genetic data or de-identifying neuroimaging data (6, 7). Ultimately, these planning discussions led to the identification of several key requirements: 1) protect participant privacy and confidentiality, 2) protect the scientific integrity of the studies, 3) release as much data as possible as soon as possible, 4) establish data use agreements that promote unhindered scientific inquiry while prohibiting malfeasance, 5) provide well-documented, standards-based metadata and data, and 6) measure the impact of data sharing activities using meaningful performance indicators.

Consistent with NIH requirements, the data sharing plan outlined the types of data to be shared, a data classification model, data ownership, access controls and permissions, data processing, security measures, release schedules, and data use requirements, among other elements.

In the interest of sharing as much data as early as possible, the decision was made to release data in two tranches. The first tranche, which included all pre-randomization data, was scheduled for release a year after study enrollment was completed. The second tranche, which includes the final study data set (screening, baseline, blinded treatment/observational, open-label), is scheduled to be released one year after the completion and database lock of the study.

### Participant and Study Partner Consent

Written informed consent was attained from study participants and their partners to allow for broad sharing of data and biospecimens collected during the study for secondary research. The development and management of consent language and materials were overseen by the regulatory sponsor, the study team, and the institutional review boards of record at each performance site.

### Data Preparation

The process of preparing data for sharing included general and data-type-specific steps focused on de-identification, linkage, formatting, and versioning. De-identification steps for all data types included removing site identifiers and masking participant and study partner identifiers, removing all free-text comment variables, and replacing dates with a set of discrete categorical visit identifiers and numeric variables denoting the number of days from consent and randomization or baseline. The HIPAA Safe Harbor method (8) was applied to de-identify all remaining variables. Neuroimaging data (MRI series: T1 - Sagittal 3D Accelerated MPRAGE, T2_star - Axial T2-Star, FLAIR - Axial T2-FLAIR, T2_SE - Axial T2-TSE with Fat Sat) were further de-identified using specialized “de-facing” methods (7). Tabular data were formatted as comma-separated values (CSV) text files. Neuroimaging data were converted from DICOM to NIfTI (Neuroimaging Informatics Technology Initiative) format. GWAS genetic data files were released using the PLINK format. Documentation outlining these processes and changes between data releases was provided with each data release to support transparency and reproducibility.

### Analysis Software

Analysis software was included in each data release to facilitate data accessibility, usability, and reproducibility of key study publication results. These software packages were implemented using open-source R statistical software and R Markdown literate programming methods.

### Study Documents

Comprehensive data dictionaries were supplied for each data file. Additional materials included methods documentation, annotated worksheets, and a primer to help investigators familiarize themselves with the documentation, data sets, and analysis software.

### Data Sharing Platforms

The first tranche of the A4/LEARN data was shared via the Laboratory for Neuroimaging’s Image and Data Archive (LONI IDA) (ida.loni.usc.edu) at the University of Southern California’s Stevens Neuroimaging and Informatics Institute. This platform serves as the datasharing repository for a large number of observational studies in neurodegenerative diseases. To further improve the findability and accessibility of the A4/LEARN pre-randomization data, a summary data set with over 500 variables was also uploaded to the Global Alzheimer’s Association Interactive Network platform (GAAIN) (www.gaain.org) that allows investigators to explore de-identified harmonized data across multiple Alzheimer’s disease cohorts. Interested investigators can also find information on how to submit data requests for full data access via the GAAIN platform.

## Results

### Pre-randomization Data

**Figure 1:**
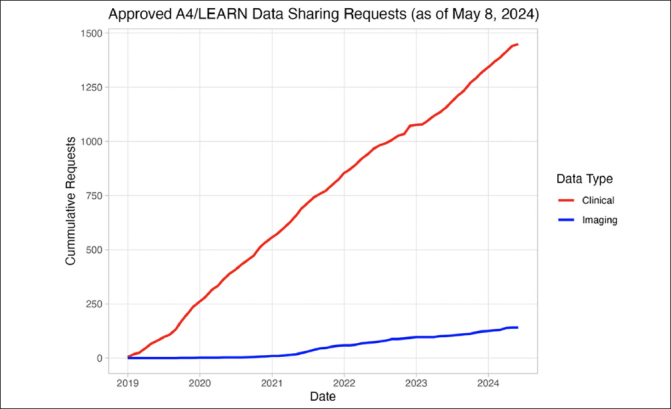
Approved A4/LEARN Data Requests (Tranche 1: Pre-randomization) (December 2018 to May 2024). 1506 data requests were submitted; 1382 (91.8%) data requests were approved; 141 approved requesters downloaded neuroimaging data.

The A4 and LEARN pre-randomization study data were released in December 2018. As of May 8, 2024, 1506 requests have been submitted by investigators and citizen scientists, of which 1382 (91.8%) have been approved (Figure 1). The approved requesters were affiliated with organizations from the University/Research Institute (n=1213, 87.8%), Biotech (n=81, 5.9%), Pharmaceutical (n=32, 2.3%), Government (n=18, 1.3%), Scanner manufacturers (n=10, 1.0%), and Other (n=95, 6.9%) sectors across 58 countries. The top five countries of origin for approved requests were: 1) United States of America (n=550, 39.8%), 2) China (n=210, 15.2%), 3) Korea (Republic) (n=73, 5.3%), 4) India (n=70, 5.1%), and 5) Germany (n=46, 3.3%).

Upon approval, requesters can access clinical data (29 files), genetic data (5 files), neuroimaging results (7 files), metadata (1 file), analysis software (1 file), and documentation (10 files). These data represent 18445 pre-randomization participant visits. Additionally, 6726 NIfTI-formatted MRI (n=1793), Amyloid PET (n=4486), and Tau PET (n=447) imaging studies are also available for download. As of May 8, 2024, a total of 847907 imaging series have been downloaded by 141 requesters (Figure 1).

To quantify the scientific impact of sharing the A4/LEARN pre-randomization data, we conducted a literature search (Google; date: May 15, 2024; search term: “‘a4 study’ alzheimer’s de-identified permission share data public-private-philanthropic partnership”) that identified 49 peer-reviewed publications that acknowledge the A4/LEARN study.

### Final Study Data

**Figure 2:**
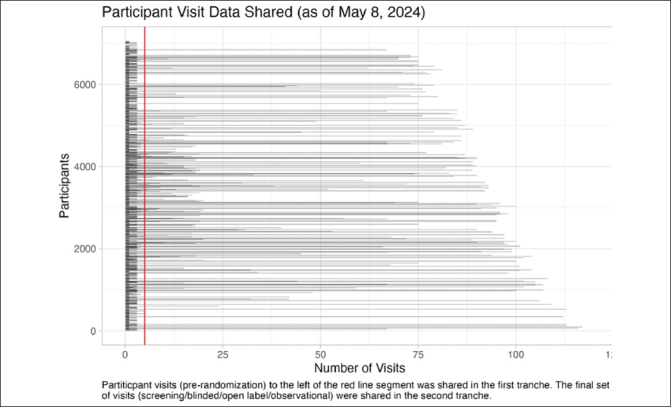
A4/LEARN Participant Visit Data Shared Participant visits to the left of the red line segment were shared in data tranche 1 (pre-randomization) (release date: December 2018). Tranche 2 (final study data) will include all participant visits (release date: June 2024).

The second tranche of final study data will be shared in June 2024. It will expand the previously shared data to include longitudinal clinical data (27 files), genetic data (5 files), neuroimaging results (9 files), clinical laboratory results (2 files), cognitive testing data (3 files), fluid biomarkers (3 files), metadata (4 files), analysis data sets (6 files), analysis software (2 files), and documentation (16 files). Coded concurrent medications, medical history, and adverse events data will be included in a future release. The magnitude of this update is extraordinary: the final study data set includes 103351 (A4=96962, LEARN=6389) participant visits, representing an increase of 560.3% in available participant visits (103351/18445) relative to the first tranche (Figure 2).

The final set of longitudinal neuroimaging studies will be released in the fall of 2024. This data set is expected to include more than 15 thousand longitudinal studies composed of 51429 NIfTI-formatted MRI (n=42858), Amyloid PET (n=6859), and Tau PET (n=1712) imaging series. Notably, the de-facing process will be extended to include all PET data (FBP: 18F-florbetapir [AV45], FTP: 18F-flortaucipir [T807, AV1451]).

## Discussion

Our initial results provide evidence supporting the feasibility and scientific utility of broad and timely sharing of Alzheimer’s disease trial data. These results are significant because although the A4 study failed to show a therapeutic benefit (1), these data are very informative for ongoing and future prevention trial designs. In many cases, negative trials have gone unpublished or have been published after long delays (9), with limited sharing of data, squandering the substantial investment of time and resources required to conduct trials. We hope these results will encourage other investigators to commit to broad and timely publication of study results and data regardless of study outcomes.

Our experience in sharing the first tranche of the A4/LEARN data demonstrates the importance of incorporating data sharing into the early stages of study planning. Preparing the first tranche of data required extensive input from multiple stakeholders and over a year of sustained effort from a multidisciplinary team of experts in biostatistics, biomarker analysis, biobanking, data management, genetics, informatics, neuroimaging, legal and regulatory compliance across multiple institutions. Informed by this experience, planning and implementation activities for the second tranche of study data began over a year before the end of the A4 study. Our ultimate goal is to conduct data collection and sharing activities concurrently by taking advantage of opportunities to introduce automation to streamline data preparation and packaging. This approach has already been implemented in ongoing studies. We plan to report on the results of this approach in future work.

While most of the effort involved in data sharing focuses on design, curation, and preparation activities, we have also identified an opportunity to explore novel platforms that offer investigators an intuitive user interface and sophisticated data exploration, visualization, and analytical capabilities. Platforms that go beyond providing basic file download capabilities by leveraging innovations in the fields of open science, natural language processing (NLP), large language models (LLMs), and distributed computing are of particular interest. We are currently evaluating platforms that may, for example, provide investigators with a chatlike interface to explore and quantify study data using natural language prompts.

### Limitations

Though our initial results are encouraging, we must also highlight some limitations. First, despite success in sharing over 5000 biospecimens, the process and methods for sharing biospecimens remain a work in progress. Our team has struggled to efficiently work through the thicket of legal and regulatory requirements necessary to transfer specimens and collect results from approved requesters, leading to delays and higher-than-expected costs.

Second, we have encountered challenges in sharing A4/LEARN whole genome sequence (WGS) data due to their magnitude (∼100 Terabytes) and increased risk to participant privacy and confidentiality. We have consulted with domain experts at NIA and the National Institute on Aging Genetics of Alzheimer’s Disease Data Storage (NIAGADS) repository at the University of Pennsylvania to offer qualified investigators access to these data. This remains a work in progress.

Finally, given the required expertise and costs of planning, preparing, maintaining, and providing ongoing administrative support for sharing large and complex trial data sets, questions remain regarding the long-term viability of these initiatives. While we are encouraged by examples of how general open science data repositories (e.g., Zenodo, OSF, Dryad) have addressed some of these challenges, extended support for IT, data management, and administrative support remain open issues. We are heartened to see a recent request for information from NIA (Notice ID: NOT-AG-24-013) that seeks to engage the AD scientific community in a dialogue on these issues.

## Conclusions

Conducting clinical trials requires the selfless contributions of many participants, study partners, and families, and the investment of substantial resources from multiple stakeholders. Maximizing the scientific utility of these commitments is not only a regulatory requirement but an ethical and scientific duty. By sharing the A4 and LEARN study data as broadly and early as possible, we have aspired to meet these obligations. The initial results presented in this article provide supportive evidence of the feasibility and scientific impact of these efforts and hint at the potential utility available in the release of the final study data. We look forward in anticipation of what discoveries the scientific community will achieve when supplied with these data.

## Notes

*Ethical Standards:* Approval from an institutional review board or ethics committee was obtained at each of the sites. All participants and their study partners provided written informed consent prior to data collection, which included consent for data sharing.
